# Distribution, abundance and diversity of the extremely halophilic bacterium *Salinibacter ruber*

**DOI:** 10.1186/1746-1448-4-15

**Published:** 2008-10-28

**Authors:** Josefa Antón, Arantxa Peña, Fernando Santos, Manuel Martínez-García, Philippe Schmitt-Kopplin, Ramon Rosselló-Mora

**Affiliations:** 1 División de Microbiología and Instituto Multidisciplinar para el Estudio del Medio Ramon Margalef, Universidad de Alicante, 03080, Alicante, Spain; 2 Helmholtz Zentrum Muenchen, German Research Center for Health and Environment, Institute of Ecological Chemistry, Neuherberg, Germany; 3 Marine Microbiology Group, Institut Mediterrani d'Estudis Avançats, (CSIC-UIB), 07190, Esporles, Spain

## Abstract

Since its discovery in 1998, representatives of the extremely halophilic bacterium *Salinibacter ruber *have been found in many hypersaline environments across the world, including coastal and solar salterns and solar lakes. Here, we review the available information about the distribution, abundance and diversity of this member of the *Bacteroidetes*.

## Introduction

During the summer of 1998, in the course of a study focused on the identification by fluorescence *in situ *hybridization (FISH) of the then uncultured square archaeon, high proportions of *Bacteria *were detected by FISH in crystallizer ponds from solar salterns [1]. Although bacterial 16S rRNA gene sequences had been previously detected in these environments [2,3], that was the first report on high abundance of potentially active *Bacteria*, for which the *candidatus *name of "Salinibacter" was proposed. Shortly after, some of these *Bacteria *could be grown in pure culture and were characterized taxonomically [4,5]. The *candidatus *species was finally classified as a new genus and species, and named as *Salinibacter ruber gen. nov. sp. nov*. In these last few years a considerable advance in the knowledge of these microorganisms has been achieved [6-8] and even its genome has been completely sequenced and annotated [9].

According to phylogenetic reconstructions based on the 16S rRNA gene [5] and on the inter-spacer region between the 16S and 23S rRNA genes [10]* S. ruber *can be affiliated with the phylum *Bacteroidetes*, being its closest related cultured organism *Rhodothermus marinus*, a thermophilic, slightly halophilic marine bacterium. The clade comprising *R. marinus *and *S. ruber *appeared as a deep branch within the phylum, placed close to the node of bifurcation of the superphylum that comprises *Bacteroidetes *and *Chlorobi *[11]. The phylogenetic position of *S. ruber *was further studied analyzing a total of 22 genes from the genome of *S. ruber *strain M31 [12]. All these genes had essential functions for the organism, were dispersed within the genome, and rendered a final alignment informative enough for phylogenetic reconstructions. Although single genes supported different topologies, the tree topology of concatenated genes was identical to that previously observed based on small subunit 16S rRNA gene analysis [12], a further confirmation of the validity of this gene for genealogical reconstruction.

This bacterium turned out to be extremely interesting for its surprisingly high similarity with haloarchaea: both types of microorganisms share the same habitat, are extremely halophilic, aerobic and heterotrophs, pigmented, maintain high intracellular potassium concentrations, have very high GC proportion in their genomes (with the exception of *Haloquadratum walsbyi*), and retinal proton pumps in their membranes. Indeed, one of the most striking features of *S. ruber *is the presence in its membrane of xanthorhodopsin [13], a retinal proton pump with a light-harvesting carotenoid antenna, that represents "the simplest electrogenic pump with an accessory antenna pigment".

Both *Salinibacter *and most of extremely halophilic *Archaea *inhabit hypersaline environments, i.e. environments with salt concentrations above that of seawater, very often close to saturation. These environments are among the most extreme on Earth since their microbiota is normally exposed to more than one stress: high salt, high radiation, some times high pressure or high pH. In particular, we have focused our studies on an artificial hypersaline environment: the solar salterns. They consist of a series of shallow ponds connected in a sequence of increasingly saline brines that are used for the commercial production of salt from seawater. During evaporation of sea water, sequential precipitation of calcium carbonate and calcium sulphate occurs, leaving a hypersaline sodium chloride brine that precipitates in ponds known as crystallizers (salinity above 30%). Although there are some other microorganisms present in low numbers, the prokaryotic community in crystallizers is dominated by dense populations of halophilic square *Archaea *(*Haloquadratum walsbyi*) and a lower proportion, from 5 to 30%, of extremely halophilic members of the *Bacteria *such as *S. ruber *[4,14] or, in some instances such as in Maras salterns (see below), *Salicola *spp. [15]. Inside the *Eukaryotic *domain, the green alga *Dunaliella *acts as the primary producer. In addition, hypersaline environments show one of the highest number of virus-like particles (VLP) reported for planktonic systems [16].

The fact that *S. ruber *shares its habitat with extremely halophilic *Archaea *together with the many "haloarchaeal-like" characteristics of this bacterium indicated that it could have experienced lateral gene transfer (LTG) from/to *Archaea*. The analysis of *S. ruber *M31 genome suggested that this was indeed the case, although the amount of genes likely involved in LGT events was more modest than expected [9]. In any case, *S. ruber *proteins, although not necessarily related to their archaeal homologues, are adapted to function at high salt and therefore have a high proportion of acidic amino acids, which yields an acidic proteome with a median isoelectric point of 5.2 [9]

Here we will focus on what we have learned during these almost ten years about the distribution, abundance and diversity of *Salinibacter *spp. For a more comprehensive review on other aspects of the biology of this bacterium, the reader is referred to the corresponding chapters in The Prokaryotes and the Bergey's Manual of Systematic Bacteriology [17,6].

## Abundance and distribution

*Salinibacter *representatives have been detected in the environment using different techniques, with different levels of sensitivity that can yield contradictory results even when applied to the same sample (some examples are given below). Therefore, one must be aware of their characteristics in order to compare results obtained using different techniques. Our group has used basically three methods for the detection of *S. ruber *and relatives in natural samples: FISH, 16S rRNA gene clone libraries and DGGE analysis, and culture. Among these three, fluorescence *in situ *hybridization, FISH, is the only method for direct quantification in natural samples as it permits the identification of single cells by means of the use of phylogenetic probes. However it has some well known limitations like problems with cell permeation, relatively high thresholds of ribosome content, accessibility to the secondary structure, etc. [18]. One of the major constraints of the technique is the database comprehensiveness, i.e. when new sequences belonging to a given group are discovered, probes should be re-evaluated and redesigned so they target the whole group (see below the example of *Salinibacter *sequences in Tuz Lake).

Second, a microorganism can be detected in environmental samples by analysis of 16S rRNA gene sequences PCR amplified from environmental nucleic acids, either by clone library construction or denaturing gradient gel electrophoresis (DGGE). DGGE is normally used for community fingerprinting as it allows the separation of different sequences of the same size on the same denaturing gel. Since they include a PCR step in which 16S rRNA genes are amplified using "specific" primers (not always so specific) and environmental DNA as template, both techniques do not provide quantitative data but, obviously, allow for the detection of sequences related to that of *S. ruber*. In the case of DGGE, there is an extra limitation since the sequences retrieved are partial and therefore almost two thirds of the phylogenetic information is missing.

Finally, the culture approach provides a source of detection and quantification despite it has important limitations derived from the different degree of culturability of the natural microbial populations. In any case, it provides important and accessible information on the isolates.

In addition, *Salinibacter *spp. have been also monitorized by means of other techniques like sulfonolipid detection [19], pigment composition analyses [20], total melting profiles, and reassociation techniques [21], among others.

**Figure 1:**
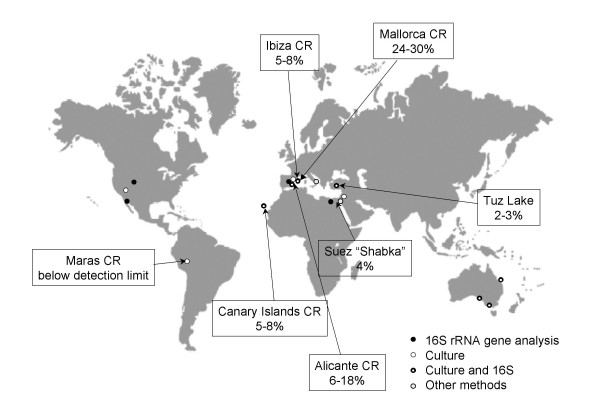
**Distribution of *Salinibacter *clones or isolates around the world, indicating the detection methods used.** For the locations were FISH data are available, the abundance of *S. ruber *is provided. CR: crystallizer.

Using all these methods, *S. ruber *or closely related bacteria has been detected all over the world (Figure 1). In Europe, for instance, *Salinibacter *representatives have been found in crystallizer pond salterns in mainland Spain (Alicante and Tarragona), Balearic (Mallorca and Ibiza) and Canary Islands. In all cases, the bacterium was detected both by culture and molecular methods, including FISH, DGGE and 16S rRNA gene clone library analysis. Additionally, the analysis by electrospray mass spectrometry of lipid extracts allowed the detection of *S. ruber *sulfonolipid signature peak at m/z 660 in a crystallizer pond in the Margherita di Savoia salterns in Italy [19].

**Figure 2:**
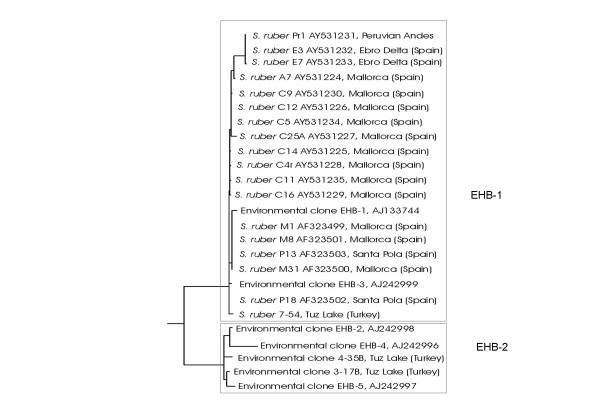
Phylogenetic maximum likelihood reconstruction based on 16S rRNA gene sequences from isolates and clones affiliated to *Salinibacter*, indicating the two phylotypes of *Salinibacter *(EHB-1 and EHB-2).

In Asia, *Salinibacter *close relatives have been found in Turkey and Israel. When analyzing the bacterial community inhabiting the hypersaline Tuz Lake in central Anatolia, Turkey [22], sequences related to *Salinibacter *dominated bacterial 16S rRNA gene clone libraries and DGGE profiles although FISH counts gave very low numbers. A close look at the new 16S rRNA gene sequences showed that many of them, although clustering with *Salinibacter *phylotypes (see below and Fig. 2), lacked the signature sequences targeted by the FISH probes. This is an example of how dependent FISH results are on the specificity of the probes. Therefore, caution must be exerted when analyzing by FISH a microbial community since, strictly speaking, probes should be always rechecked against 16S rRNA gene sequences retrieved from the samples being analyzed. In Israel, although *S. ruber *could not be detected by culture-independent methods in water samples from Eilat salterns, it could be readily isolated [23].

In Africa, sequences related to *S. ruber *have also been retrieved from water and sediments from three different soda lakes in the Wadi An Natrum depression in Egypt [24]. We also detected *S. ruber *by FISH in a water sample taken from a shabka in Suez (Fig. 1).

In the Andean Maras salterns, in Peru, *S. ruber *could be easily isolated in culture from brine samples taken in different years although it could not be detected either by FISH or by analyzing 107 bacterial 16S rRNA gene clones [15]. In fact, the bacterial community there was dominated by members of the class *Proteobacteria*, and specially representatives of the recently classified *Salicola marasensis *[15]. Finally, during the last Halophiles meeting, the presence of *S. ruber*-related sequences has also been reported for other locations in America, such as the Great Salt Lake in Utah (oral presentation by C. D. Litchfield), in the atalassohaline Andean Lake Tebenquiche in Northern Chile (oral presentation by C. Pedrós-Alió), and Guerrero Negro (oral presentation by S. Sabet) salterns in Baja California, Mexico.

Both clones and isolates very closely related to *S. ruber *have been recently found in crystallizer ponds from three different salterns in Australia (Dickson Oh and Mike Dyall-Smith, personal communication): one at Dry Creek, South Australia; another in Lara, Victoria, and a third in Bajool, Queensland (Fig. 1). Most interestingly, some of these isolates correspond to the so-far uncultured (see below) cluster EHB-2.

**Figure 3:**
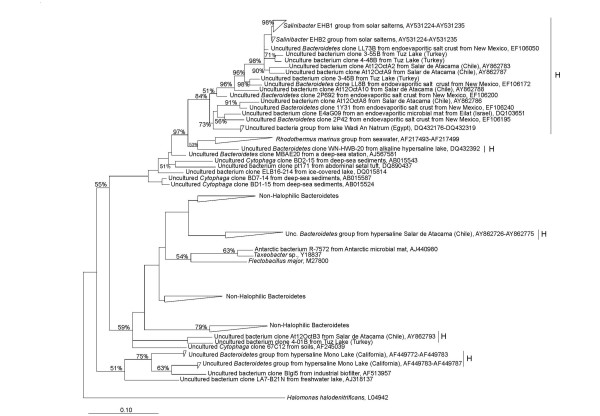
**Phylogenetic reconstruction based on 16S rRNA gene sequences from isolates and clones affiliated to *Bacteroidetes*.** The tree is based on the results of a maximum likelihood analysis and shows quarter puzzling support values. Bar, 10% estimated sequence divergence. Accession numbers of individual sequences are shown on the tree. Sequences retrieved from hypersaline environments are labelled with H. Modified from [22].

Apart from sequences clustering with *S. ruber *phylotypes EHB-1 and EHB-2 (from Extremely Halophilic Bacteria 1 and 2 [4], Figure 3), *Bacteroidetes *sequences more distantly related to *S. ruber*, were very abundant in 16S rRNA gene libraries constructed with DNA extracted from the different layers of an endoevaporite (crystallized gypsum-halite matrix in near-saturated salt water) from saltworks in Guerrero Negro, Mexico [25]. In addition, partial 16S rRNA gene sequences with similarities of less than 92% to *Salinibacter *have been retrieved from biofilms colonizing Mayan monuments in Uxmal (Mexico) [26]. Also, similar gene sequences were found in clone libraries obtained from a hypersaline endoevaporite microbial mat from a pond of 20% salinity in Eilat salterns in Israel [27]. These sequences were most abundant in the green layer of the mat. Finally, 16S rRNA gene sequences that could represent distinct novel lineages within the radiation of the genus *Salinibacter *have been recovered from evaporite crusts in brine pools at the Badwater site in Death Valley National Park, California [28]. Bacteria with similarities between 93 and 94% with *S. ruber *16S rRNA gene have been isolated from these samples [28].

As illustrated by all these examples, *S. ruber *and relatives have been detected by different methods in a wide variety of environments. In some case, the bacterium is abundant, as directly demonstrated by FISH or other methods, or has been retrieved only by cultivation. The relevance of the finding of *Salinibacter *in a given environment depends on the technique used for its detection: it is possible, as with the example of Maras salterns, that the bacterium is a very minoritary component of the community or, as seen in Mallorca salterns, is a very abundant component, easily detectable. Thus, depending on the analyzed environment, *Salinibacter *spp. can be either one of the most abundant, and most likely ecologically relevant organisms, or be part of what has been called "the seed bank" [29]. According to Pedrós-Alió [29], most abundant taxa in a given environment would be the "core" species that would be maintained through active growth and fuel carbon and energy flows. This group would be accessible through molecular techniques such as 16S rRNA gene clone library analysis. In addition, there would be a diverse assemblage of rare taxa, or "seed bank", that "will seldom be retrieved" by this molecular approach but could occasionally be recovered by cultivation. In our examples of Mallorca and Maras samples, *S. ruber *would be core and seed bank, respectively. One thus may hypothesize that in a given environment *S. ruber *could also change its status as it becomes more or less abundant. However, the preliminary data we have obtained so far with crystallizer ponds from solar salterns indicate that although the bacterium experiences changes in numbers along the year, those are not very dramatic, at least under "normal" environmental conditions (unpublished results).

The abundance of *Salinibacter *spp. has been thoroughly measured only in a few hypersaline environments and therefore, we do not have a clear picture of how abundant this bacterium is at a global scale. In the places analyzed, *Salinibacter *spp. ranges form 2 to 30% (see Figure 1) although in some cases, like Tuz Lake, this value is clearly an underestimation since the FISH probes used did not target the whole assemblage of *Salinibacter *sequences. In Santa Pola salterns, *Salinibacter *spp. was detected only in ponds with salinities above 22.4%. In fact, the numbers detected by FISH increased with salinity (from 3.5 to 12% in three ponds of 25, 31.6 and 37% total salts) [4]. However, direct proof of the activity of *Salinibacter *spp. in the highest salinity (37% total salts) ponds has not been obtained so far. In fact, Gasol et al. [30] found evidences that above 32% salinity all the prokaryotic activity was carried out by haloarchaea in the same ponds where *S. ruber *accounted for up to 18% of the DAPI counts. This observation was based on the assumption that *Salinibacter *was not inhibited by taurocholate, which is a potent haloarchaeal inhibitor. In a recent work [7], Elevi Bardavid and Oren have shown that taurocholate does not inhibit aminoacid synthesis by *Salinibacter*. Therefore, the function of *Salinbacter *in most saturated crystallizers remains unkown.

## Diversity at 16S rRNA gene level

The first step taken after the detection by FISH, ten years ago, of abundant members of the *Bacteria *domain in a crystallizer pond, was the analysis by DGGE of the bacterial community in the systems. Two bands were obtained, corresponding to two partial bacterial 16S rRNA gene sequences, named as EHB-1 and EHB-2. The analysis of a 16S rRNA gene library from the same sample allowed the retrieval of the complete EHB-1 and 2 sequences, that shared an identity of 97.6%. FISH quantification with specific probes indicated that EHB-1 was the dominant bacterial phylotype in Santa Pola Salterns (about 5 times more abundant than EHB-2 detected cells). Shortly after, isolates related to these sequences were retrieved from crystallizers in Mallorca and Santa Pola. All theses isolates, a total of 5, and the rest (around 200) that we have obtained since then from these and other salterns in mainland and inland Spain, clustered with EHB-1 (Figure 2). When more *S. ruber *isolates were obtained from salterns in Israel, Peru, and Turkey, all of them belonged also to EHB-1. Only very recently, Dickson Oh and Mike Dyall-Smith have been able to retrieve a member of the EHB-2 group, most likely a new species of the genus *Salinibacter*, in pure culture (personal communication).

Since the discovery of *Salinibacte*r, many other sequences affiliating with the phylum *Bacteroidetes *have been retrieved from hypersaline environments around the world including, for instance, athalassohaline environments such as the Lake Chaka (32.5% salinity) in Northwestern China [31], and lakes from Los Andes with a wide range of salt concentrations and compositions [32]. The obvious question to ask is whether these sequences form a monophyletic group; in other words, whether there is a group of extremely halophilic *Bacteroidetes *that have evolved from a common ancestor or the sequences retrieved from hypersaline environments are scattered across this phylum. To address this question, we constructed [22] a tree with complete sequences of *Bacteroidetes *retrieved from hypersaline and other environments, including cultured and non-cultured microorganisms (Figure 3). Indeed, there is a big group of sequences, which includes *Salinibacter*, that all come from high salt places (labelled with H in the figure), although there are also "high salt sequences" scattered across the tree. However, there is a group that includes *Salinibacter *and is formed only by sequences retrieved from hypersaline environments such as endoevaporitic salt crust from New Mexico, water samples from lakes in Salar de Atacama (Chile) or an endoevaporitic microbial mat from Eilat (Israel). This group may represent additional genera, different from *Salinibacter*.

## Comparison of *S. ruber *strains isolated from different locations: clues for biogeography

As previously shown for extremophiles such as the archaeon *Sulfolobus *[33] and thermophilic *Cyanobacteria *[34], the extreme conditions and geographical isolation of the environments from which we have isolated *S. ruber *are an optimal scenario for observing allopatric speciation processes in prokaryotes. However, our initial studies based on genomic fingerprinting by pulsed field gel electrophoresis and randomly amplified polymorphic DNA analyses, although indicating a certain trend, did not provide clear evidence of geographical discrimination for *S. ruber*. [10]. In addition, and contrary to what was observed with fluorescent pseudomonads [35], internal transcribed spacer (ITS) sequences were not suitable for ascertaining biogeographical similarity since all these strains had identical or very similar ITSs. In order to compare the geographical genetic discrimination of extremophiles evidenced by Whitaker et al. [33], we studied a subset of 10 strains using multilocus sequencing analysis (MLSA). For these, 8 housekeeping genes phylogenetically informative were PCR amplified and sequenced. In contrast to what was observed for thermophilic *Archaea*, this analysis did not show a clear geographical segregation of the analyzed strains [36]. Obviously, this finding is not meaning that there are no genetic differences between the strains just that, in our case, MLSA cannot be correlated with their geographical origins.

As an alternative to the genetic studies, we recently [36] undertook a metabolomic study focused to understanding of the metabolic similarities occurring among isolates of the same location. We studied a collection of 28 *S. ruber *strains isolated from 7 different locations in the world (Mallorca, Alicante, Tarragona, Ibiza, Israel, Canary Islands and Peru). These locations corresponded to three main geographical areas (Mediterranean: 10 strains, Atlantic: 13 strains, and Peruvian: 5 strains).

**Figure 4:**
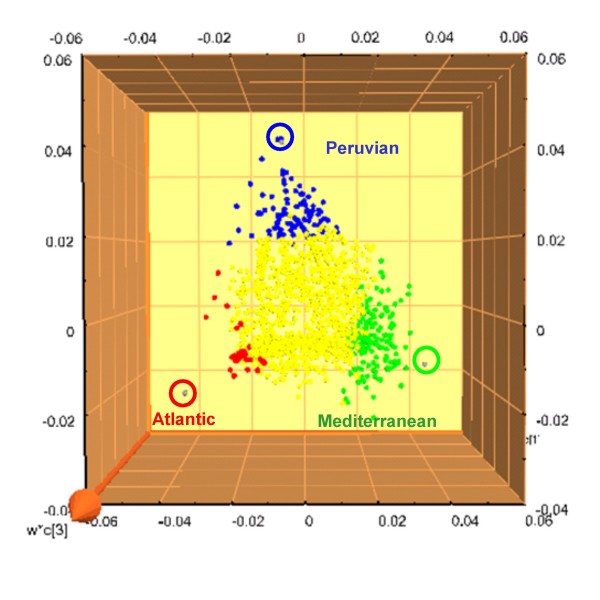
**Plot correlating the 2099 m/z values of known elementary composition to the geographical origins of isolation of the analyzed *S. ruber *strains.** The m/z values having a high correlation with geographical origin are highlighted in red, blue or green while the nondiscriminating masses are represented in yellow.

The 28 strains were then analyzed using ion cyclotron resonance Fourier transform mass spectrometry (12T ICR-FT/MS), a mass spectrometry method that currently provides the highest available molecular resolution. All strains were grown at the same time under the same conditions and extracts from their supernatants and the cellular soluble and insoluble fractions were analyzed. The common peaks for all extracts (i.e. the core metabolome) consisted of 2550 single m/z compounds while there were 6323 peaks not common to all extracts (or discriminative metabolites). The analysis of presence or absence of individual metabolites did not alone reveal clear geographical trends. However, when the relative intensity of every individual peak (the concentration of every metabolite) was introduced in the considered data, a multivariate analysis revealed "statistically significant differences between the different samples" ([36], Figure 4). Samples from different origins had thus different levels of expression of certain metabolites, and these differences could be correlated with the origin of isolation of the strains. The metabolites responsible for the geographic differentiation could be putatively identified as having aliphatic structures depleted in oxygen, such as fatty acids and terpenoids, that are generally associated with cell membranes.

**Figure 5:**
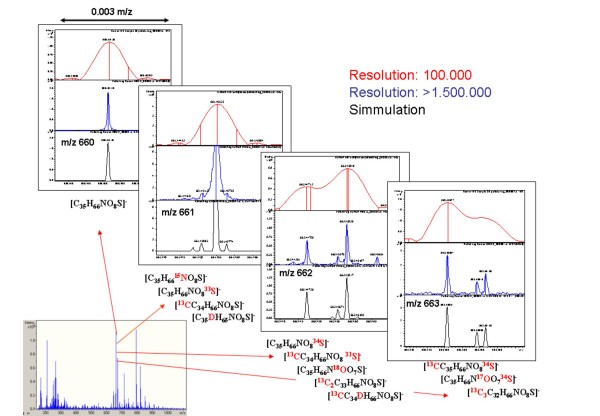
Detailed spectra of *S. ruber *sulfonolipids in negative-mode electrospray ICR-FT/MS.

We could also identify the presence of typical sulfonolipids previously described for *S. ruber *strain M31 [19]. Our analysis showed that besides the M31 sulfonolipid, *S. ruber *may contain at least 9 additional sulfonolipids, with variations in their elementary composition, saturation and side chain structure (Figure 5). In addition, some sulfonolipids specific for Atlantic strains were also found.

All these results indicated that there is a metabolic diversity among *S. ruber *strains that can be correlated with geographical patterns. Indeed, the three groups of strains analyzed came from three clearly different ecosystems. Although all the strains had been isolated from crystallizer ponds, the strains from Maras salterns (the Peruvian group) came from an inland saltern located at more than 3000 meter above sea level, in which the prokaryotes were around one or order of magnitud less abundant than in coastal crystallizer ponds, most likely due the lower concentration of nutrients (these salterns are fed by saline spring water) and the low environmental temperatures. *S. ruber *were very minority members of the microbial community of these salterns, as explained above, and showed very low genomic diversity [37]. On the other side, Mediterranean strains came all from coastal crystallizer ponds exposed to rather high temperatures in the summer, in which prokaryotic numbers were above 10^7 ^cells/ml, with high nutrient concentration (the salterns are fed with coastal sea water, that is concentrated more than 10 fold in the crystallizers), in which the *S. ruber *assemblage is abundant and diverse [37]. Atlantic strains were all isolated from solar salterns in the Canary island, that would correspond to intermediate conditions between Peruvian and Mediterranean strains. It is not surprising that strains that have to cope with so different environmental conditions display different metabolomes. Indeed, comparison of the metabolomes indicated that the largest differences were found between Peruvian and Mediterranean strains [36].

## Future research

Now that we know that the genus *Salinibacter *is widespread, diverse and abundant, we can address more questions relevant to its ecology and activities in the environment. For instance, why is *Salinibacter *so often present in hypersaline environments although is always outnumbered by *Archaea*? How do they interact? Do they compete for nutrients or kill each other with halocin-like molecules? Under which circumstances, if any, could *Salinibacter *outgrow haloarchaea? What is the role of phages in maintaining the equilibrium between extremely halophiles of both Domains. What is the diversity of *Salinibacter *beyond 16S rRNA? Do different *S. ruber *types compete among them? Most of these questions are at this moment unanswered although we are starting to get insight into some of them. We know, for instance, that the response of *Salinibacter *and haloarchaea to environmental stresses such as high radiation, is different (Santos et al., unpublished results). We have also found that certain haloarchaea can inhibit *Salinibacter *growth (Nercessian et al., unpublished; Elevi-Bardavid and Oren, Halophiles 2007 meeting) and that different strains of *S. ruber *can show antagonistic effects (Peña et al., unpublished). In addition, the diversity of *S. ruber *seems to be much higher than 16S rRNA analysis indicated: for instance, we isolated 45 new strains (López-Pascual et al., upublished results) from the very same crystallizer where the type strain was isolated 9 years before; despite the fact that all these strains had identical 16S rRNA genes, they displayed 22 different PFGE patterns, none of them corresponding with that of the type strain.

In future, we will focus our research in these aspects and follow looking for *Salinibacter *spp. and other new extremely halophilic microorganisms in hypersaline environments across the Earth. Hopefully, within the next ten years we will be able to answer some of the questions that these last ten years of research have allowed us to ask.

## Competing interests

The authors declare that they have no competing interests.
